# Supplementary material for: Septin 9 Orients the Apico–Basal Polarity Axis and Controls Plasticity Signals
Source: Cells. 2023 Jul 9;12(14):1815. doi: 10.3390/cells12141815 (PMC10377970; doi:10.3390/cells12141815)
Supplement: Supplementary file 1 [file cells-12-01815-s001.zip › cells-2472823-supplementary.pdf]

cells MDPI

Supplementary

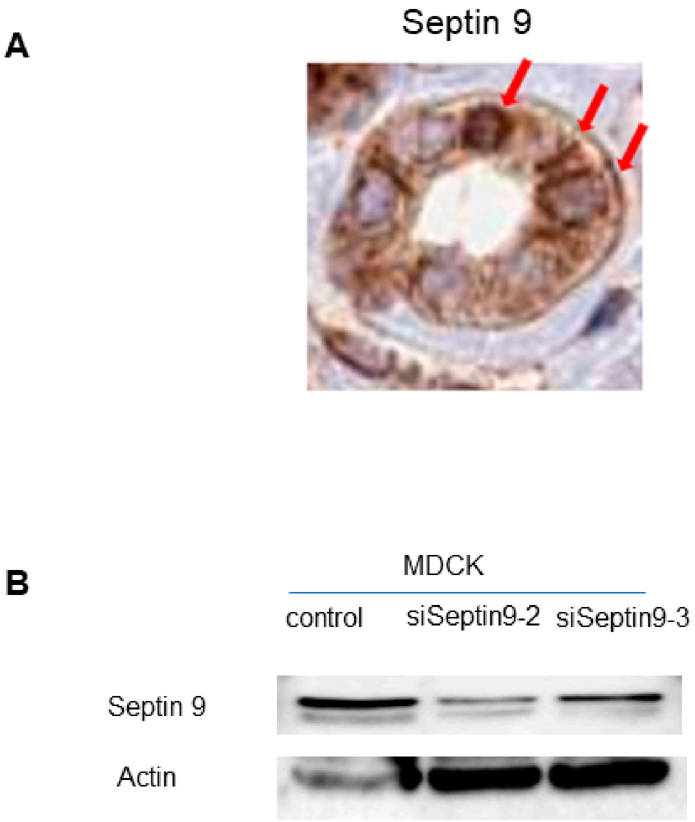

Figure S1. Supplementary data on endogenous Septin 9 and its siRNA.

A. Endogenous Septin 9 staining in the tissue of a human kidney, acquired from the Protein Atlas, *HPA042564, Female, age 41, Kidney (T-71000), Normal tissue, NOS (M-00100), Patient id: 2530.*

B. MDCK cells were transfected with scrambled siRNA (control) or with two septin 9 siRNA (siSeptin9 #2 and siSeptin9#3) for 24 h, then the medium was changed to continue grown on plates for 3 days. Cells were lysed and analyzed by western blot for the expression of septin 9 protein.

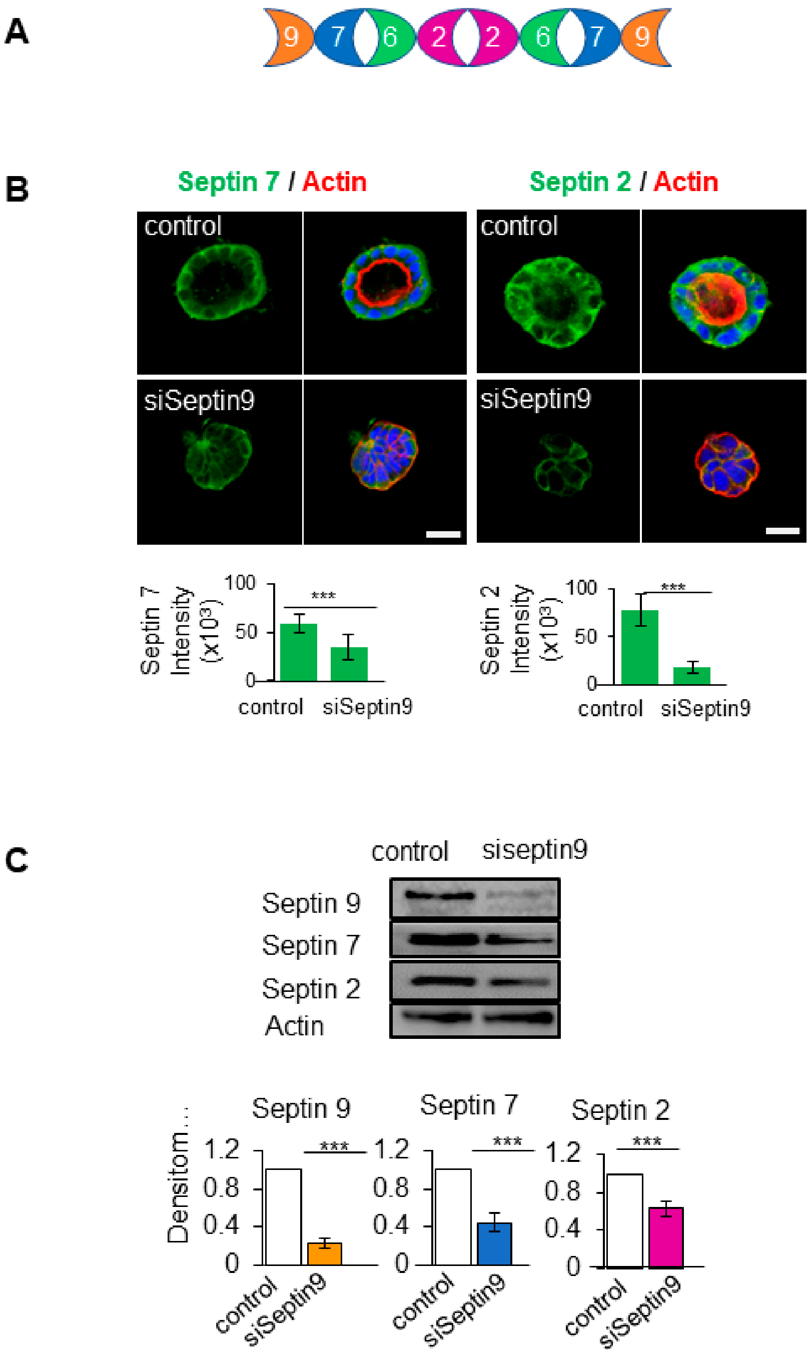

Figure S2. Septin 9 regulates the expression of septin 2 and septin 7 and their localization at the BM

A. Schematic representation of the interaction between septin 9 and other septins.

B. MDCK cells were transfected with scrambled siRNA (control) or with septin 9 siRNA for 24 h and plated on Matrigel for 4 days to form cysts, and then stained for septin 7 (green), septin 2 (green) and actin (red). A single confocal section through the middle of a cyst is shown. Scale bar 10 µm. Quantification of the fluorescence intensity of each protein expression: septin 2 (green), and septin 7 (green).

C. MDCK cells were transfected with scrambled siRNA (control) or with septin 9 siRNA (siSeptin9) for 24 h, then the medium was changed to continue grown on plates for 3 days. Cells were lysed and analyzed by western blot for the expression of septin 9, septin 7 and septin 2 proteins.

Data information: Data concern at least two replicates and cysts (n>10) for 3D staining, and three for immunoblotting. The statistical values are means ±s.e.m. Student's t-test was used. ***$P < 0.001$.

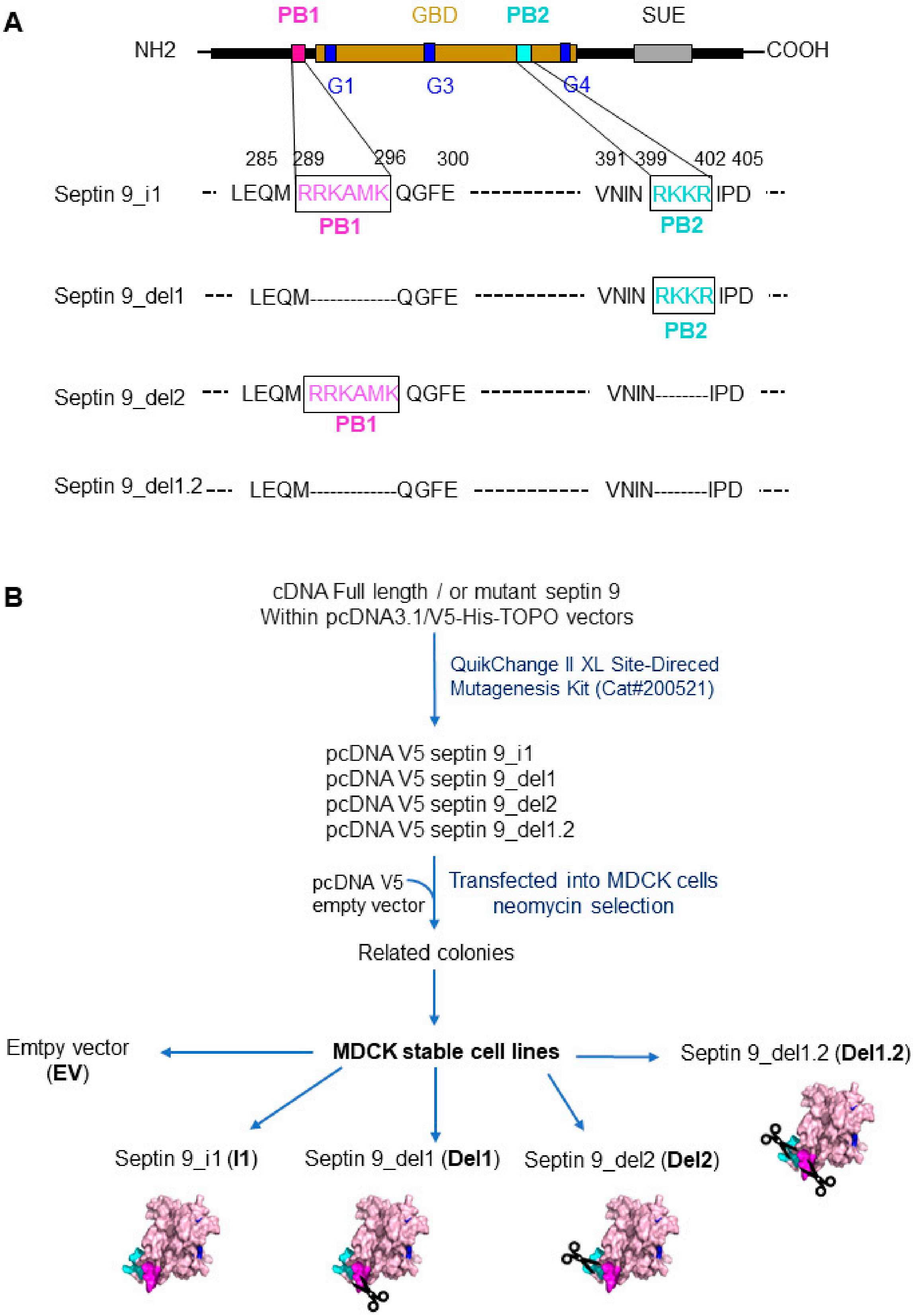

Figure S3. Constructs used during the study

A. Septin 9 domain organization and its polybasic domain 1(PB1) (RRKAMK=R^{289}RKAMK^{296}), and polybasic domain 2 (PB2) (RKKR=R^{399}KKR^{402}). We showed multiple alignments of septin 9_ i1, septin 9_del1, septin 9_del2 and septin 9_del1.2.
B. Experimental flowchart for making plasmids and the related stable MDCK cell lines, including septin 9_empty vector (EV), septin 9_i1(Il), septin 9_del1(Del1), septin 9_del2(Del2) and septin 9_del1.2 (Del1.2) in 3D structure.

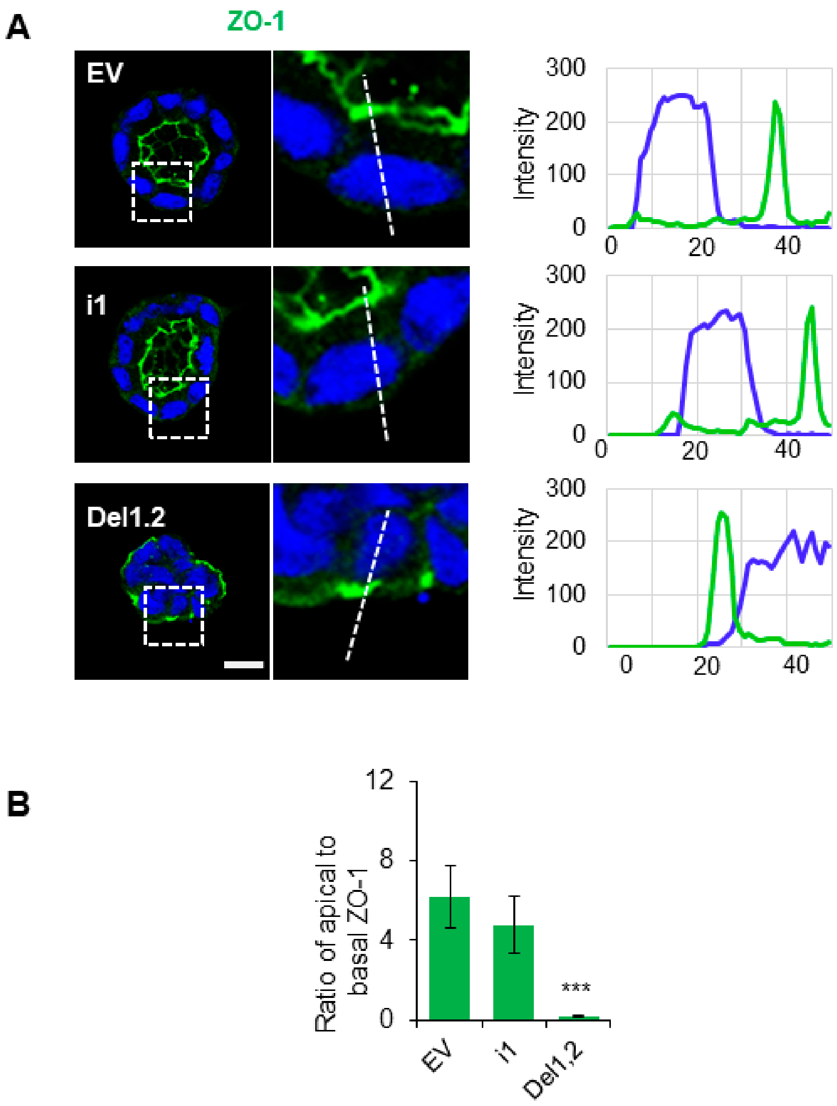

Figure S4. The deletion of septin 9_i1 PB domains impacts tight junctions

A. MDCK cells expressing EV, i1 and del1.2 were plated on Matrigel for 4 days to form cysts, and then stained for ZO-1 (green). A single confocal section through the middle of a cyst is shown. Scale bar 10 µm. Line profiles showing ZO-1 distribution from the basal to the apical areas, obtained using Image J are presented.

B. Quantification of ZO-1 distribution: the ratio of maximal fluorescence intensity at the apical side versus maximal fluorescence intensity at the basal side, ***P <0.001.

Data information: Data concern at least two replicates and cysts (n>10) for 3D staining. The statistical values are means ±s.e.m. Student's t-test was used. ***$P < 0.001$.

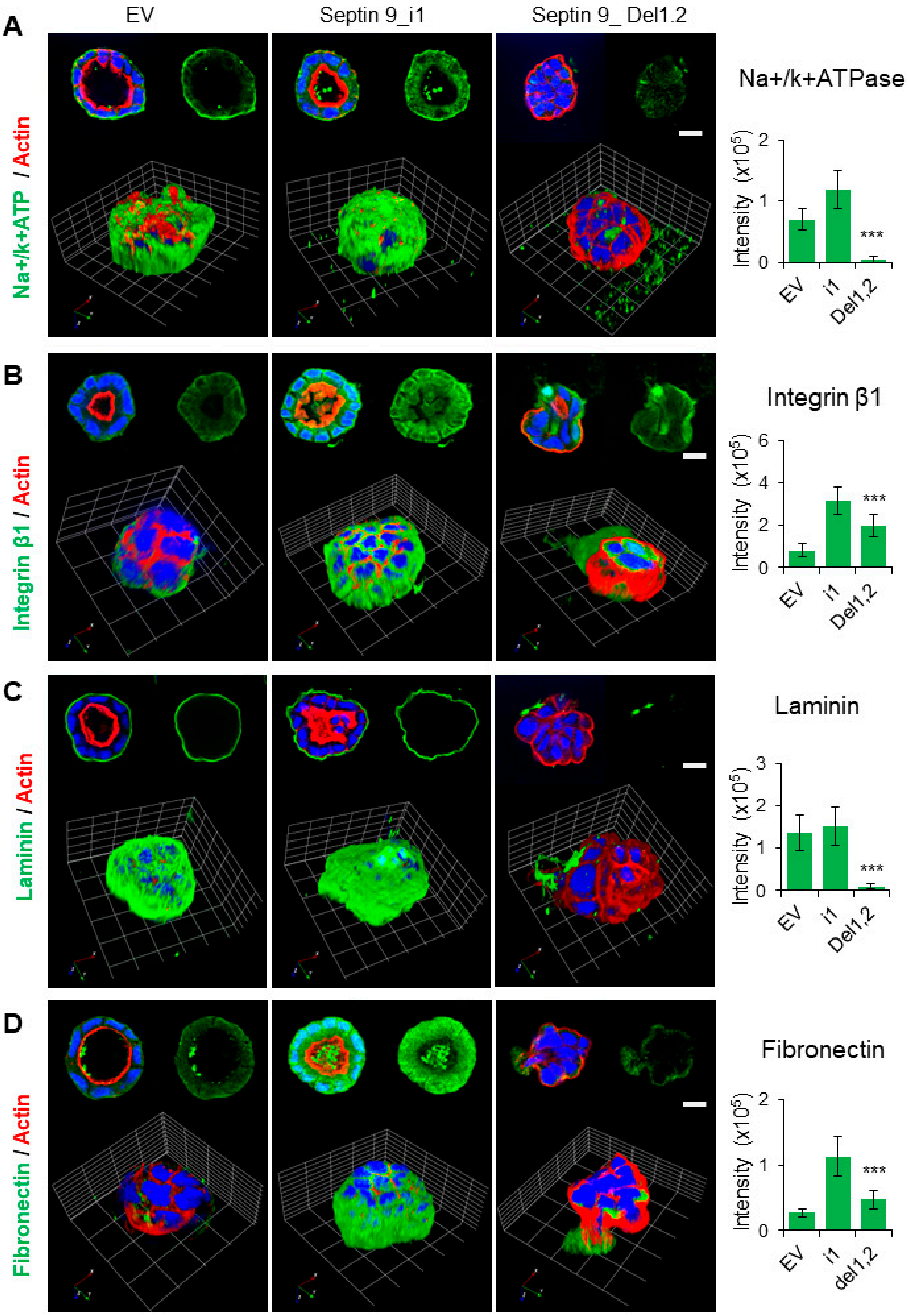
A
EV
Septin 9_i1
Septin 9_ Del1.2
Na+/k+ATP / Actin
Na+/k+ATPase
Intensity (x10^5)
2
1
0
***
EV
i1
Del1,2
B
Integrin β1 / Actin
Integrin β1
Intensity (x10^5)
6
4
2
0
***
EV
i1
Del1,2
C
Laminin / Actin
Laminin
Intensity (x10^5)
3
2
1
0
***
EV
i1
Del1,2
D
Fibronectin / Actin
Fibronectin
Intensity (x10^5)
2
1
0
***
EV
i1
del1,2

Figure S5. The deletion of septin 9_i1 PB domains impacts cell-ECM adhesion

A-D. MDCK cells expressing EV, i1 and del1.2 were plated on Matrigel for 6 days to form cysts and were stained for Na+/K+ ATPase (green) and actin (red), integrin β1 (green) and actin (red), laminin (green) and actin (red), and fibronectin (green) and actin (red). A single confocal section through the middle of a cyst is shown. Scale bar 10 μm. The 3D reconstructions of all cysts are presented. Quantification of the fluorescence intensity of Na+/K+ ATPase, integrin β1, laminin, and fibronectin is shown. The data are means ±s.e.m. Student's t-test was used. ***p < 0.001.

Data information: Data concern at least two replicates and cysts (n>10) for 3D staining. The statistical values are means ±s.e.m. Student's t-test was used. ****P* < 0.001.

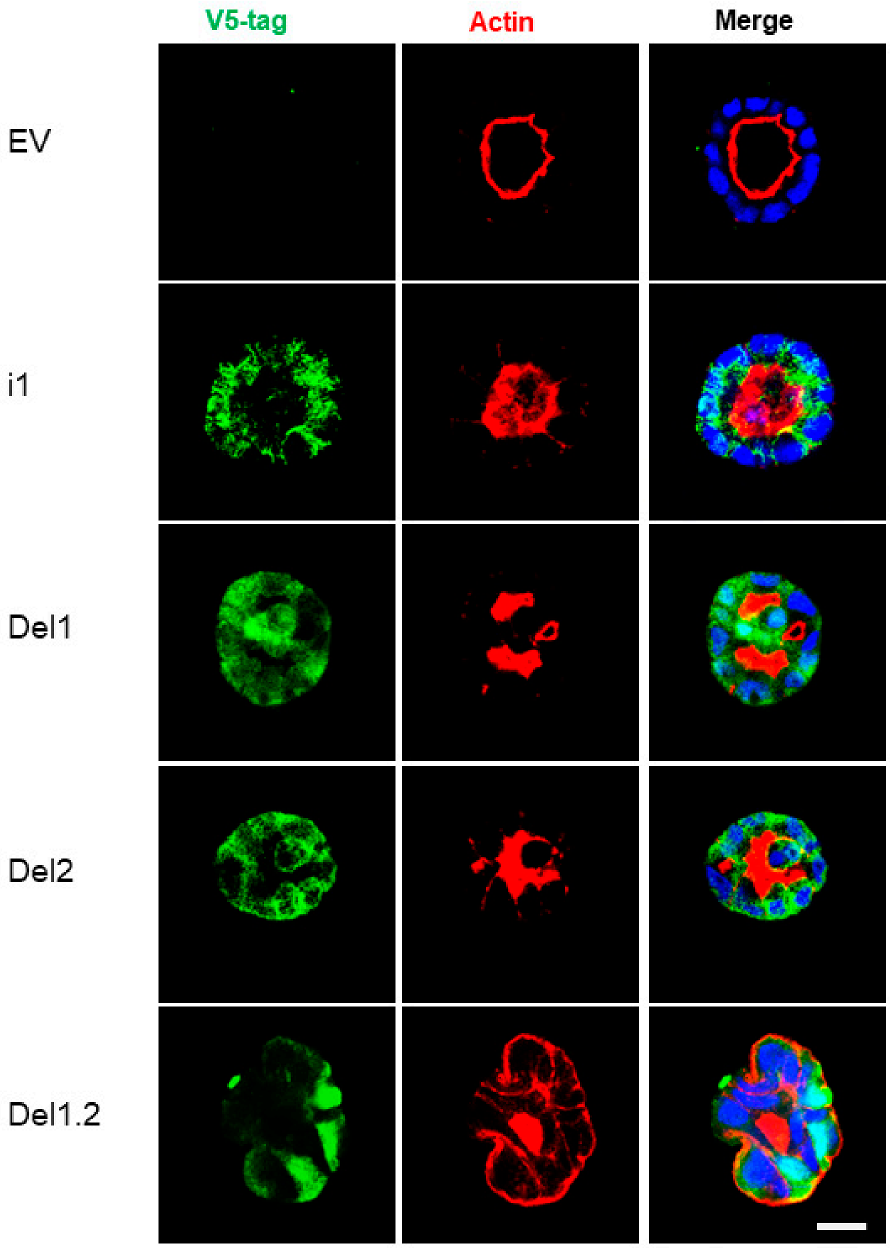

Figure S6. Septin 9-V5 tag expression in MDCK stable cell lines at day 6

MDCK cells expressing EV, i1, del1, del2, and del1.2 were plated on Matrigel for 6 days to form cysts and stained for septin 9- V5 tag antibody (green), and actin (red). A single confocal section through the middle of a cyst is shown. Scale bar 10 µm.

Data information: Data concern at least two replicates and cysts (n>10) for 3D staining.

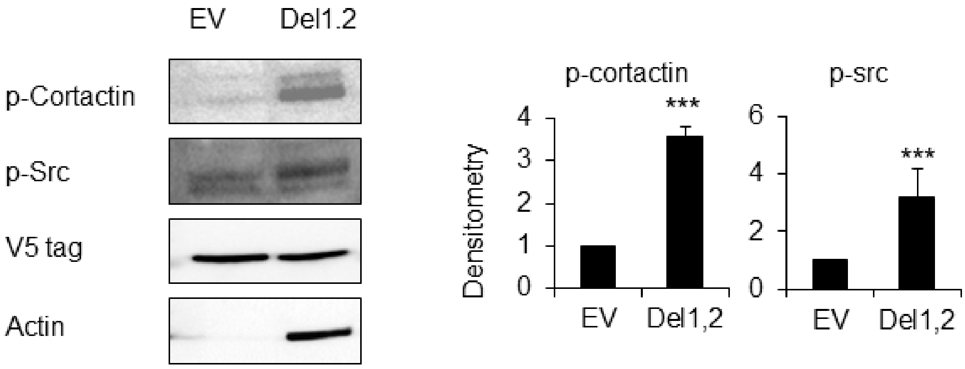

Figure S7. The deletion of Septin 9_i1 PB domains increases the activation of p-cortactin and p-src

Immunoblotting of p-cortactin and p-src protein in MDCK Septin 9_EV and Septin 9_Del1.2.

Data information: Data concern three for immunoblotting. The statistical values are means ±s.e.m. Student's t-test was used. ***$P < 0.001$.

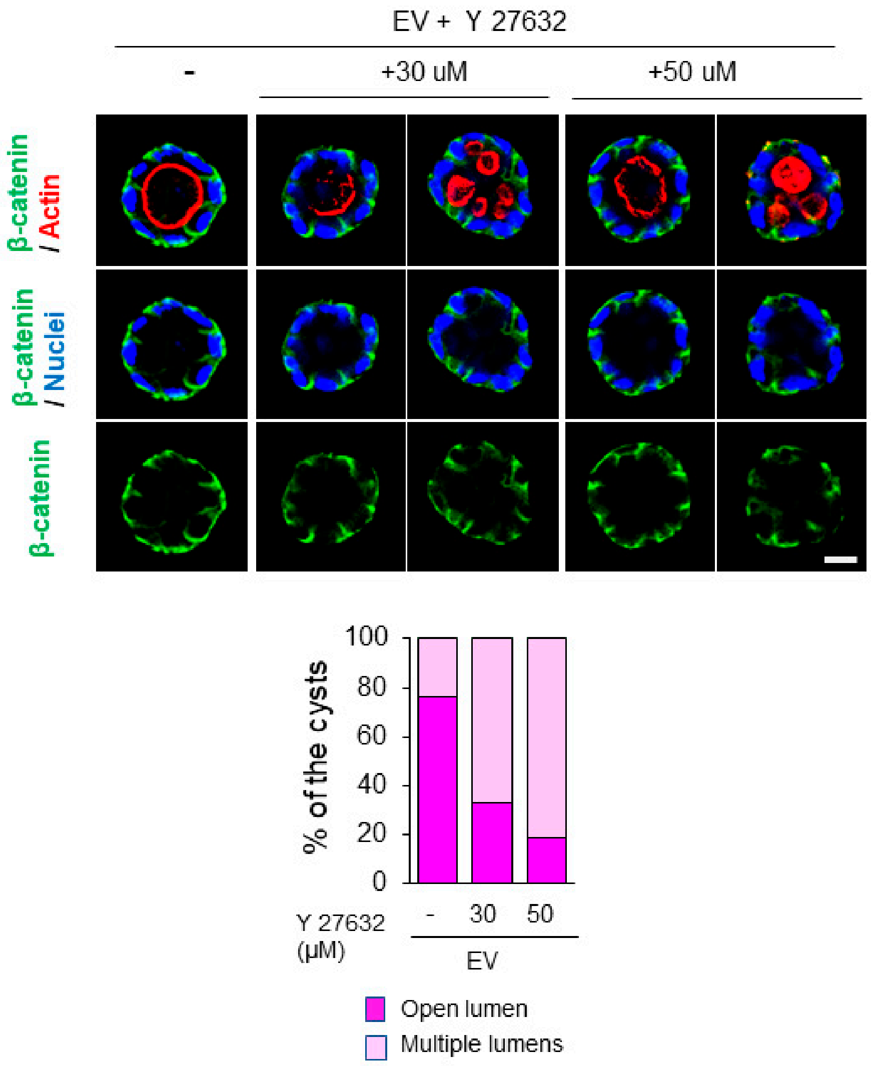

Figure S8. The treatment of septin 9_EV cysts with Y27632 rescues polarity

MDCK septin 9_EV cells were plated on Matrigel for 6 days and treated with 30 µM and 50 µM of Y27632. All cysts were stained with β-catenin (green) for the basolateral membrane, actin (red) for the apical surface and Hoechst (blue) for nuclei. Representative confocal images are show in a single merge and nuclei. Quantification of polarized and inverted polarity phenotypes in septin 9_EV cysts.

Data information: Data concern at least two replicates and cysts (n>100) for 3D staining.

For Fig 2C

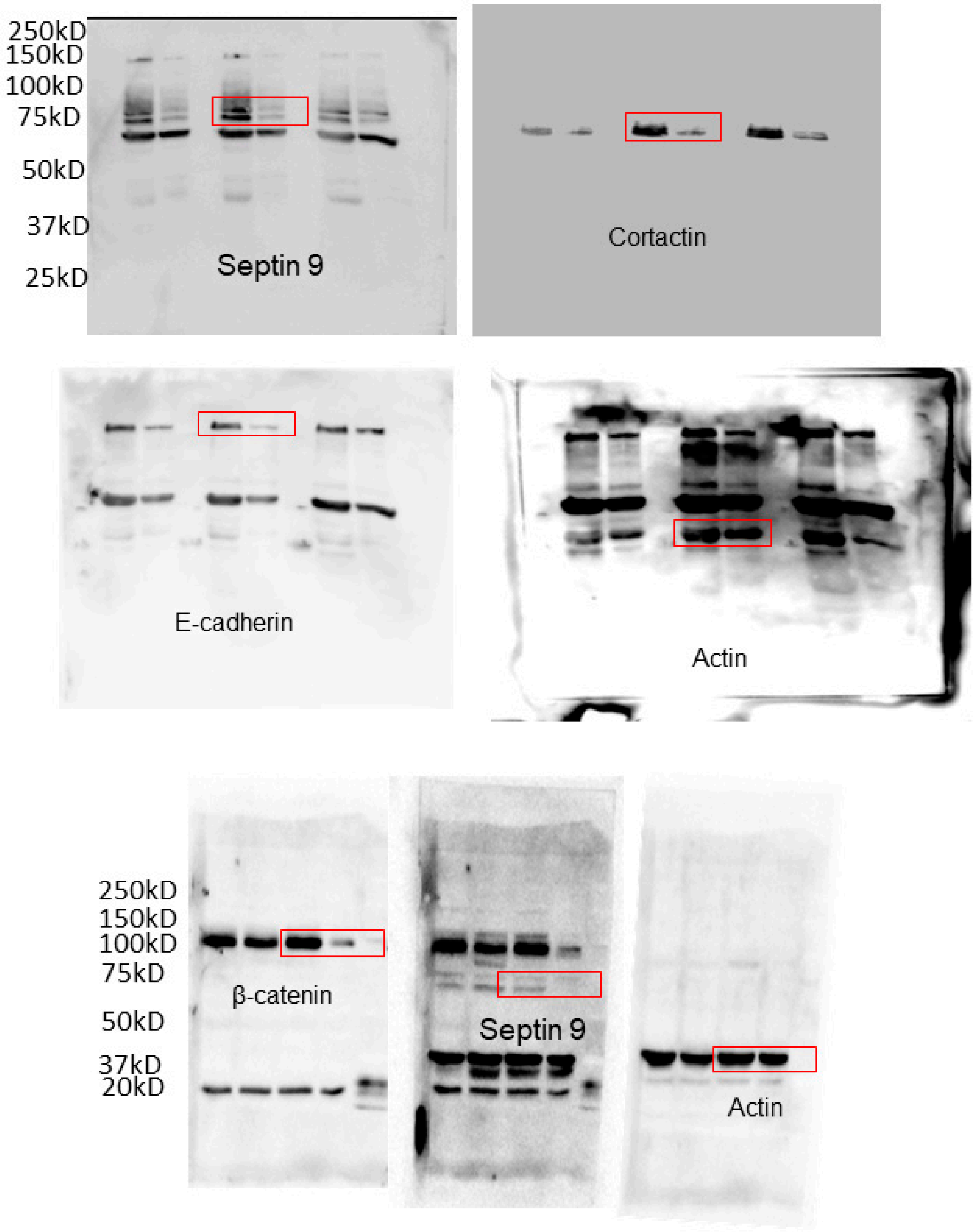

Figure S9. Raw data of immunoblotting related to the Fig. 2C.

For Fig 3B

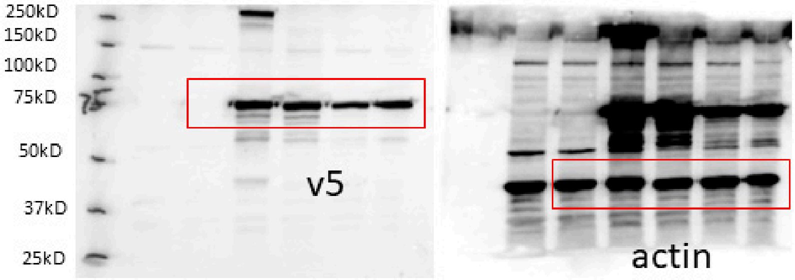

For Fig 3E

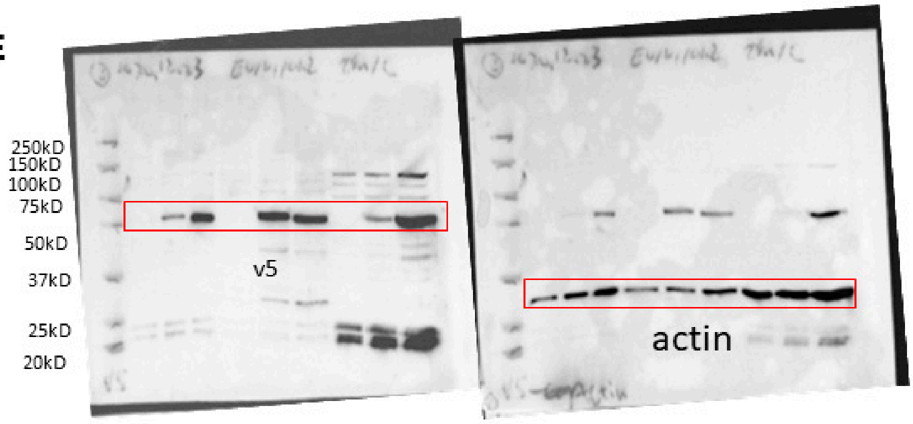

For Fig 6C

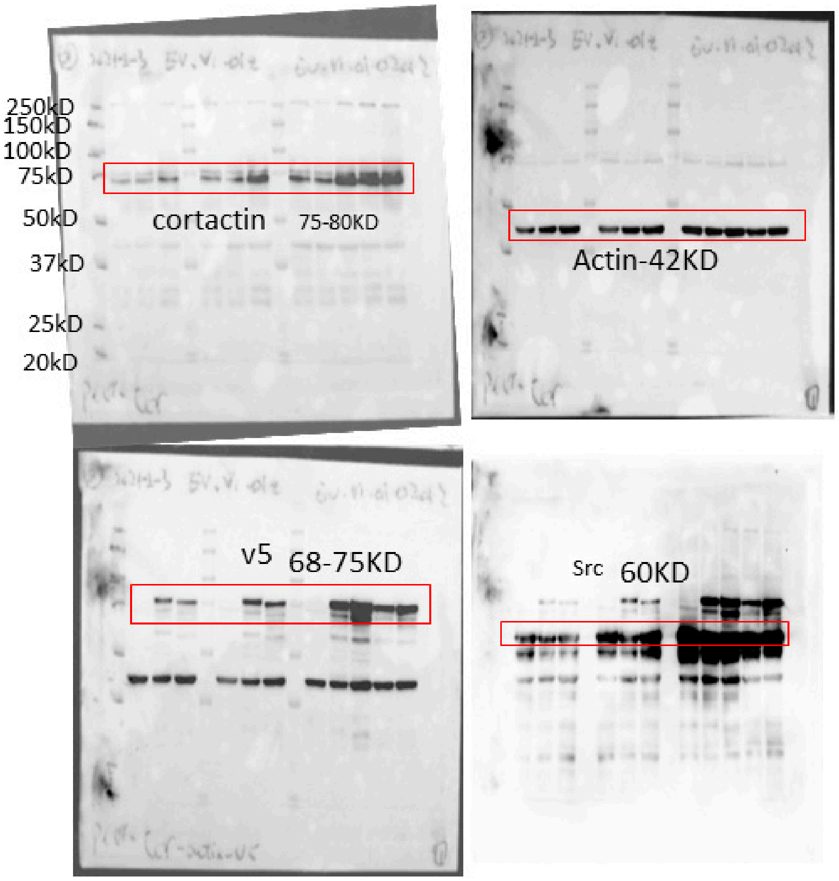

Figure S10. Raw data of immunoblotting related to the Fig. 3B, Fig. 3E, and Fig. 6C.

For Fig S2C

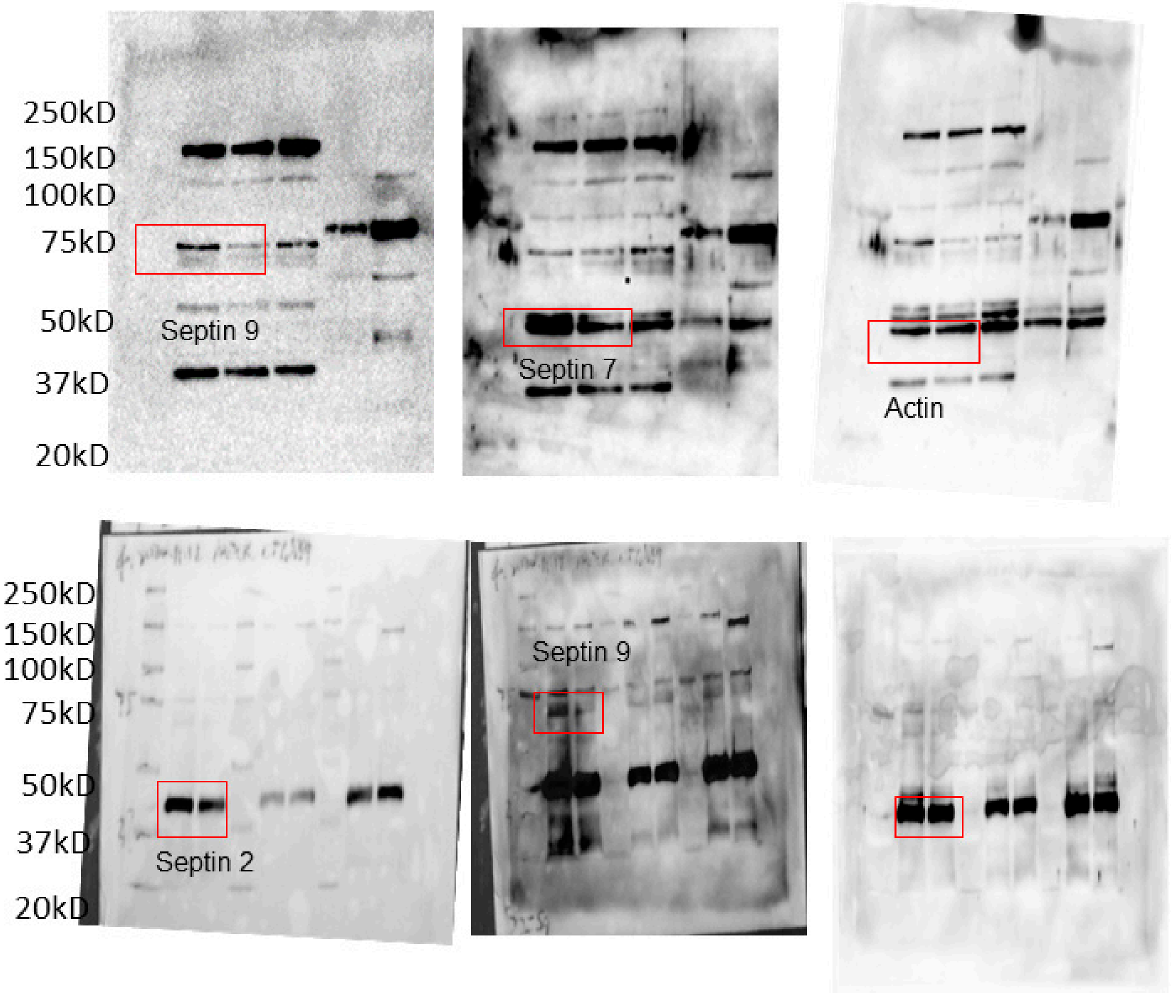

Figure S11. Raw data of immunoblotting related to the Fig. S2C.

Table s1. The sequence for the primers

Primer name	Forward sequence	Reverse sequence
Canis_SEPTIN9	GTC CAC TGC TGC CTC TAC TTC A	GGA CGA TGT TGA CCA CCT TGC T
Canis_Vimentin	GCC ATC AAC ACC GAG TTC AA	GGA AGC GCA CCT TGT CGA T
Canis_N-cadherin	CAA CTT GCC AGA AAA CTC CAG	ATG AAA CCG GGC TAT CAG CTC
Canis_ZEB1	CAA GGT GGC CAT TCT GTT AT	CTA GGC TGC TCA AGA CTG TAG
Canis_TGF-β1	GGC CAC CAT TCA TGG CAT GA	CGT GTC CAG GCT CCA AAT GT
Canis_GAPDH	CAT CAC TGC CAC CCA GAA G	CAG TGA GCT TCC CGT TCA G

Table S1. Primer sequences.
